# Supplementation of *Abelmoschus manihot* Ameliorates Diabetic Nephropathy and Hepatic Steatosis by Activating Autophagy in Mice

**DOI:** 10.3390/nu10111703

**Published:** 2018-11-07

**Authors:** Hwajin Kim, Theodomir Dusabimana, So Ra Kim, Jihyun Je, Kyuho Jeong, Min Cheol Kang, Kye Man Cho, Hye Jung Kim, Sang Won Park

**Affiliations:** 1 Department of Pharmacology, Institute of Health Sciences, Gyeongsang National University School of Medicine, Jinju 52727, Korea; hwajin1@gmail.com (H.K.); odomy2020@gmail.com (T.D.); candylll@naver.com (S.R.K.); jeri1984@naver.com (J.J.); khjeong@gnu.ac.kr (K.J.); hyejungkim@gnu.ac.kr (H.J.K.); 2 Department of Convergence Medical Sciences, Institute of Health Sciences, Gyeongsang National University Graduate School, Jinju 52727, Korea; kmc8572@hanmail.net; 3 Department of Food Science, Gyeongnam National University of Science and Technology, Jinju 52725, Korea; kmcho@gntech.ac.kr

**Keywords:** *Abelmoschus manihot*, autophagy, diabetic nephropathy, mitochondrial fission-fusion, steatosis

## Abstract

Diabetic nephropathy (DN) is a diabetic complication marked by albuminuria and a decline of the glomerular filtration rate. Diabetic kidneys are defective in the autophagy process and mitochondrial function and their enhancement of activity alleviates the pathology. In this paper, we developed a mouse model of DN by a combined treatment of a high-fat diet and streptozotocin after unilateral nephrectomy and supplementation with flower or leaf extracts of *Abelmoschus manihot* (AM) were tested. The preventive effects of the extracts on DN pathology and changes on autophagy and mitochondrial proteins were investigated. DN mice showed a significant increase in fasting blood glucose, plasma creatinine, blood urea nitrogen, and urinary albumin levels. Periodic acid–Schiff and Sirius red staining of the diabetic kidney presented a significant change in glomerular and tubular structures that was associated with podocyte loss and fibrotic protein accumulation. These changes were attenuated by AM extract treatment in DN mice. In addition, hepatic injury, proinflammatory cytokines, and lipid accumulation were decreased by AM extracts in DN mice. As a protective mechanism, AM extracts significantly increased the expression of proteins by regulating autophagy and mitochondrial dynamics, which potentially prevented the kidney and liver from accumulating pathogenic proteins and dysfunctional mitochondria, which alleviated the progression of DN.

## 1. Introduction

Diabetic nephropathy (DN) is a leading cause of end-stage renal disease. It affects both type I and II diabetics and is clinically marked by proteinuria and a decline of the glomerular filtration rate. Early stage DN exhibits glomerular hypertrophy, mesangial matrix expansion, and basement membrane thickening. Advanced DN exhibits nodular glomerulosclerosis, mesangiolysis, and tubulointerstitial fibrosis [1]. Control of blood glucose and blood pressure or the renin-angiotensin system (RAS) inhibition can slow, but not prevent, disease progression. DN has become a worldwide health problem. If not treated properly, patients require renal replacement therapy including life-long dialysis or kidney transplantation [2].

Autophagy is a conserved cellular process that degrades misfolded protein aggregates and damaged organelles via the lysosomal pathway and reconstitutes cellular components. It is essential to maintain cell homeostasis under starvation as well as during pathogenesis of metabolic diseases such as diabetes [3]. Autophagy activity is regulated through nutrient-sensing signaling pathways and is altered in diabetes by metabolic stress [4,5]. Podocytes have a glomerular filtration barrier function and insufficient autophagy in podocytes was observed in patients with diabetes and massive proteinuria [6]. The impaired autophagy activity contributes to DN pathogenesis and restoration of autophagy activity may be a promising therapeutic target of DN [7,8].

Mitochondria are cellular energy-producing organelles needed for cellular metabolic homeostasis. They are highly dynamic organelles that frequently change their shape, number, and cellular distribution. Mitochondrial fission is regulated by the activity of dynamin-related protein-1 (DRP-1) and fusion requires mitochondrial inner and outer membrane proteins, optic atrophy-1 (OPA-1), and mitofusin-2 (MFN-2), respectively [9]. The mitochondrial quality is also well-controlled by the activation of parkin-mediated or autophagy receptor-mediated mitophagy [10]. Hyperglycemia increases reactive oxygen species production through mitochondrial fission [11]. Thus, restoration of mitochondrial dynamics may delay the DN progression prior to inflammatory, apoptotic, or profibrotic signaling cascades.

Dakpul is an annual plant in Korea and its starchy substance in roots has been used to make hanji (Korean paper). It was formerly considered a species of *Hibiscus* but is now classified in the genus *Abelmoschus*. Thus, its scientific name is *Abelmoschus manihot* (AM) [12]. Huangshukuihua has been used for traditional medicine in China and its scientific name is also AM. The morphology of these two plants looks similar but differs from their appearance in the flower, leaf, and seed. Thus, Dakpul and Huangshukuihua are considered subspecies of AM [12]. AM is distributed in tropical areas especially in Asia and the Pacific Islands and its flowers and leaves are edible and exhibit medicinal properties. AM has traditionally been used for treating inflammation, pain, urinary infection, and chronic bronchitis due to its anti-inflammatory, anti-viral, anti-bacterial, and wound healing activities [13]. The Huangkui capsule (HKC), which is a Chinese traditional medicine extracted from AM, has been approved by the China State Food and Drug Administration (Z19990040) for the treatment of nephritis [14]. The bioactive components of AM include isoquercitrin, hibifolin, myricetin, quercetin-3-O-d-glucoside, quercetin, hyperoside, and gossypetin [15]. However, most studies were performed by using AM extracts from flowers and not leaves. Therapeutic effects have not been investigated for extracts from Dakpul versus Huangshukuihua. Natural plant materials often differ in their effective components depending on the cultivation area [16].

In this study, we investigated the effect of the extracts from the flower and leaf from Dakpul or Huangshukuihua on DN pathology and examined the molecular mechanisms, particularly on autophagy and mitochondrial regulation. For the first time, we determined the effects of extracts not only in the kidney but also in the liver of DN mice. We concluded that all extracts tested attenuated renal and hepatic injury possibly by activating autophagy and mitochondrial function in DN mice.

## 2. Materials and Methods

### 2.1. Preparation of Plant Extract

The Dakpul and Huangshukuihua were grown (Sancheong, Gyeongnam, Republic of Korea) and prepared as follows. The flowers and leaves were collected, air-dried, and pulverized to a fine powder. The pulverized flowers or leaves (1 g) were extracted with 20 mL of 70% aqueous ethanol at 70 °C for 5 h. This process was repeated twice and the combined extracts were concentrated at 60 °C in a vacuum condenser and lyophilized at −70 °C. The powder and extract preparation was performed by Kye Man Cho under the standard protocol [17]. The powered extracts were dissolved in distilled water for oral administration.

### 2.2. Animals, Surgery, and Treatment

Male C57BL/6 mice (7-weeks-old) were purchased from Koatech Co. (Pyeongtaek, Republic of Korea) and maintained in the animal facility at Gyeongsang National University. All animal experiments were approved by the Institutional Board of Research at Gyeongsang National University and were performed in accordance with the National Institutes of Health guidelines for laboratory animal care. Mice were housed with an alternating 12 h light/dark cycle and were provided with water and standard chow ad libitum. 

We developed a DN model by combining unilateral nephrectomy (UN), a high-fat diet (HFD), and streptozotocin (STZ) in C57BL/6 mice. It is an accelerated type 2 diabetic nephropathy model (<4 weeks). We treated the extracts at early stages of DN for delaying the progression of DN. 

Mice were habituated for 1 week and subjected to UN. The renal artery, renal vein, and ureter of the left kidney were ligated and removed. UN mice in general show no avert phenotype without a significant risk affecting the kidney such as hyperglycemia, hypertension, or kidney toxin. After 2 days, the mice were fed a normal chow diet (control, *n* = 8) or an HFD (60 kcal % fat, Research Diets, Inc., New Brunswick, NJ, USA). After 3 weeks, STZ (100 mg/kg) was intraperitoneally injected to HFD-fed mice (*n* = 40) and confirmed the levels of blood glucose (DN, 366 ± 18 dL/mg versus control, 101 ± 6 dL/mg) 48 h after STZ treatment. The blood glucose levels were also measured 24 h before sacrifice. DN mice were treated with vehicle (water) and flower (F) or leaf (L) extracts from Huangshukuihua (H) or Dakpul (D) (denoted as vehicle, HF, HL, DF and DL, *n* = 8 each) at a dose of 100 mg/kg/day by oral gavage for 5 weeks. The mice were weighed weekly and food intake was measured twice per week. Fasting blood glucose levels were measured from the tail vein by using an Accu-Chek glucometer (Roche Diagnostics, Mannheim, Germany). Upon sacrifice, the right kidney was collected and the weight was measured. After cutting the kidney in half, each half was snap-frozen in liquid nitrogen for long-term storage at −80 °C or fixed in 10% formalin. The left and median lobes of liver were collected, a part of the left lope was fixed in 10% formalin, and the rest of the liver tissues were snap-frozen in liquid nitrogen for-long term storage at −80 °C. All blood and urine samples were centrifuged at 3000× *g* for 20 min at 4 °C and all supernatants were stored at −80 °C.

### 2.3. Biochemical Assays 

Blood was collected from an inferior vena cava using a heparinated syringe and plasma was separated by centrifuging at 3000× *g* for 15 min. Plasma alanine aminotransferase (ALT) and aspartate aminotransferase (AST) activities were measured by using commercial assay kits from the IVDLab (Uiwang, Republic of Korea) and a spectrophotometer (Shimadzu UV-1800 spectrophotometer, Tokyo, Japan). Plasma creatinine (pCr) was measured by a direct colorimetric Jaffe method. Each sample was mixed with a reagent of 50 mM sodium phosphate containing 50 mM sodium borate, 4% SDS, and picric acid, incubated for 30 min, and then Abs1 (at 510 nm) was measured. After adding 60% aqueous acetic acid for 5 min, Abs2 (at 510 nm) was measured. The difference in Abs1 and 2 was used for calculation of pCr. Blood urea nitrogen (BUN) was measured by using commercial assay kits from the IVDLab and a spectrophotometer. Urine was collected from each mouse housed in a metabolic cage (Jeungdo Bio & Plant Co., Seoul, Republic of Korea) for 16 h before sacrifice. The urinary volume of each sample was measured, centrifuged at 2000× *g* for 10 min to precipitate sediment, and the supernatant was transferred into a sterile tube and stored at −80 °C. Urinary albumin and Cr were determined by colorimetric assay kits purchased from Abcam (Cambridge, MA, USA). Hepatic and plasma triglyceride (TG) levels were also measured by using a triglyceride colorimetric assay kit (Cayman, Ann Arbor, MI, USA).

### 2.4. Hematoxylin and Eosin (H&E), Periodic Acid–Schiff (PAS), and Sirus Red Staining

Fixed tissues were embedded in paraffin and the blocks were sectioned at 5 μm. For histological analyses, liver sections were stained with H&E (Sigma-Aldrich, St. Louis, MO, USA) and kidney sections were stained with Periodic acid-Schiff (PAS), according to a standard protocol. Sirius Red staining was used for visualization of collagen fibers following a standard protocol. Deparaffinized sections were incubated in Picro-Sirus Red solution (Abcam) for 1 h, rinsed twice with acetic acid solution, and dehydrated in absolute alcohol. Lastly, it was mounted for microscopy. Images were captured by using a CKX41 light microscopy (Olympus, Tokyo, Japan). 

### 2.5. WT1 Immunohistochemical Staining

Kidney sections were boiled in citric acid buffer (10 mM, pH 6.0) for 20 min for antigen retrieval. The sections were blocked in 10% normal horse serum and incubated with a primary antibody (1:100 diluted) against Wilms’ Tumor Protein 1 (WT1) from Boster Biological Technology (Pleasanton, CA, USA) overnight at 4 °C. The sections were incubated with biotinylated secondary antibody (Vector Laboratories, Burlingame, CA, USA) for 1 h at room temperature. The sections were incubated in avidin-biotin-peroxidase complex solution (ABC solution; Vector Laboratories) for 30 min and then developed using a 3,3′-diaminobenzidine (DAB) Peroxidase Substrate Kit (Vector Laboratories) for 30 s. The sections counterstained with hematoxylin were analyzed by using a CKX41 light microscope (Olympus). The number of stained nuclei was counted from 10 images of 200× microscopic fields per kidney section from each group.

### 2.6. Western Blot Analysis

Tissues were homogenized in ice-cold RIPA buffer with protease inhibitors (Thermo Fisher Scientific, Waltham, MA, USA), sonicated, and incubated for 20 min on ice. After centrifugation, the supernatant was transferred to a clean tube and protein concentration was determined by using a Bio-Rad protein assay kit (Bio-Rad, Hercules, CA, USA). The protein samples were separated by SDS-PAGE and were transferred to polyvinylidene fluoride (PVDF) membranes. After blocking with 5% skim milk, the membranes were incubated with primary antibodies against autophagy-related protein (ATG) 5 and 12, IκB(α), p-IκB(α), LC3B, Nucleoporin p62 (p62), sirtuin-1 (SIRT-1) (Cell Signaling Technology, Danvers, MA, USA), DRP-1 (Santa Cruz Biotechnology); MFN-2 (Abcam), and OPA-1 (BD Biosciences, San Diego, CA) in blocking the solution at 4 °C overnight. The membranes were incubated with horseradish–peroxidase-conjugated secondary antibodies (Bio-Rad) at RT for 1 h and the protein bands were detected with ECL substrates (Bio-Rad). The ChemiDoc XRS+ System (Bio-Rad) was used to analyze the density of each protein band. Monoclonal antibody against mouse β-actin (1:10,000, Sigma-Aldrich) was used as a loading control.

### 2.7. Reverse Transcription and Quantitative PCR

Total RNA was extracted when using Trizol (Thermo Fisher Scientific) and was converted into cDNA with the RevertAid Reverse Transcription System (Thermo Fisher Scientific), according to the manufacturer’s protocol. Quantitative PCR (qPCR) was performed on the CFX Connect Real-Time PCR System by using iQ SYBR Green Supermix (Bio-Rad). Relative mRNA levels were normalized to that of GAPDH. The primers used are listed as follows (forward, f; reverse, r). Nphs1(f) 5′-GGTGGAAGGGTCGACAGTTAAGCT-3′, Nphs1(r) 5′-CTCGTACTCCGCATCATCGCTGA-3′; Nphs2(f) 5′-CCGTCTCCAGACCTTGGAAATACC-3′, Nphs2(r) 5′-GGACACATGGGC-TAGACTGCTTAG-3′; TNF-α(f) 5′-CATATACCTGGGAGGAGTCT-3′, TNF-α(r) 5′-GAGCAATGACTCCAAAGTAG-3′; IL-6(f) 5′-CCAATTCATCTTGAAATCAC-3′, IL-6(r) 5′-GGAATGTCCACAAACTGATA-3′; IL-10(f) 5′-GGGAAGACAATAACTGCAC-3′, IL-10(r) 5′-TGAAAGAAAGTCTTCACCTG-3′. qPCR was performed with initial denaturation at 94 °C for 5 min and the cycling conditions were 45 cycles of 10 s at 95 °C, 10 s at 60 °C, and 30 s at 72 °C. The analysis was relatively quantified to GAPDH and averaged the all delta cycle threshold (Ct) values in each sample by using the Ct method. Experiments were conducted in duplicate to ensure amplification integrity.

### 2.8. Statistical Analysis

Statistical differences among the groups were determined with one-way analysis of variance (ANOVA) and was followed by Bonferroni post-hoc analysis by using the GraphPad Prism 5.0 software (GraphPad Software Inc., La Jolla, CA, USA). The values are expressed as the mean ± SEM. A *p* value < 0.05 was considered statistically significant.

## 3. Results

### 3.1. AM Extracts Improved Renal Infiltration Function in DN Mice

**Figure 1:**
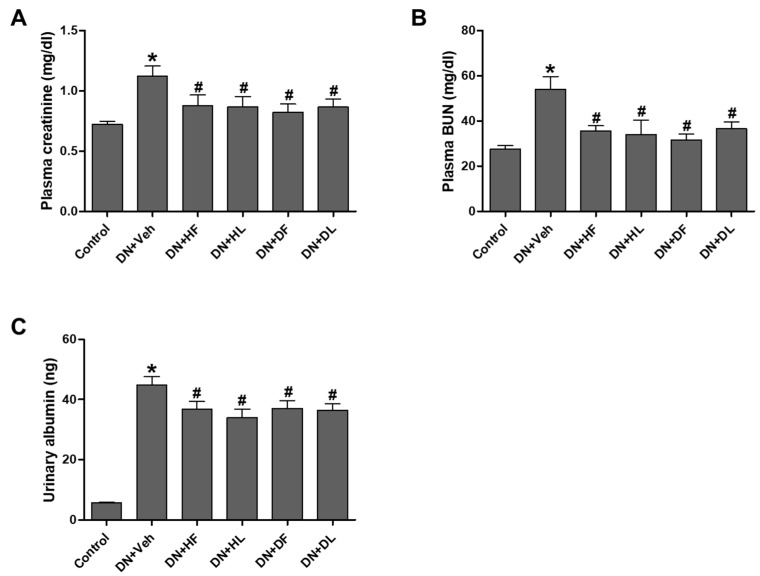
AM extract treatment reduced renal injury in diabetic nephropathy (DN) mice. C57BL/6 mice were subjected to unilateral nephrectomy and fed a high fat diet for 3 weeks and then were injected with streptozotocin to generate DN mice. For 5 weeks, DN mice were treated with flower or leaf extracts from Dakpul or Huangshukuihua (abbreviated as DF, DL, HF, or HL). Blood and urine samples were collected from control, vehicle-treated or extract-treated DN mice. Plasma levels of creatinine (**A**), blood urea nitrogen (**B**), and urinary albumin levels (**C**) were measured. The data are presented as the mean ± SEM. * *p* < 0.05 versus control, # *p* < 0.05 versus DN mice.

**Figure A1:**
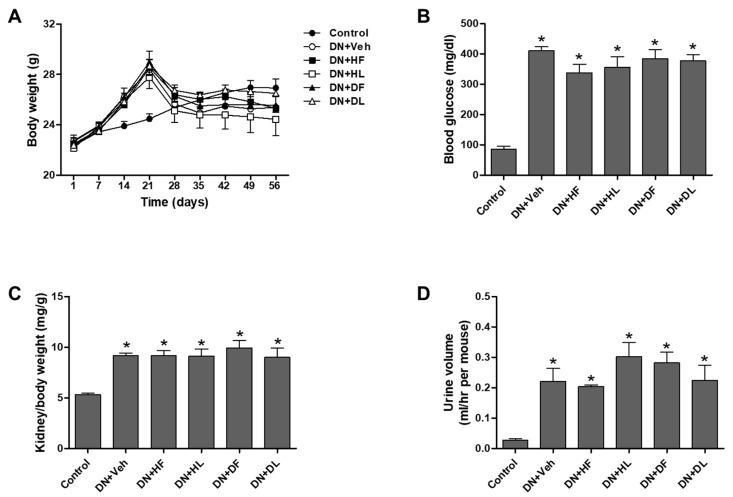
Effect of AM extracts on body metabolic parameters of DN mice: body weight (**A**), fasting blood glucose (**B**), kidney-to-body weight ratio (**C**), and urine volume (**D**). The data are presented as the mean ± SEM. * *p* < 0.05 versus control group, # *p* < 0.05 versus DN+Veh group.

To examine the effects of Dakpul and Huangshukuihua on improving kidney function in DN, mice were orally administered with each extract from flowers or leaves for 5 weeks and body metabolism and renal injury were measured. The mice treated with flower or leaf extracts from Huangshukuihua and Dakpul were denoted as HF, HL, DF, and DL. DN mice showed an increase of body weight after HFD feeding for 3 weeks and then a slight reduction after STZ injection. However, there was no change in weight from the extract treatment (Figure A1A). The levels of food intake were also not changed by the extract treatment in DN mice. We found a slight decrease in blood glucose levels in extract-treated DN mice and compared to vehicle-treated DN mice, but it was not statistically significant (Figure A1B). To assess kidney function, the levels of pCr, BUN, and urinary albumin were measured. The increased pCr and BUN levels in DN mice were significantly reduced in the extract-treated DN mice (Figure 1A,B). The urinary albumin levels were also reduced by the extract treatment in DN mice (Figure 1C). The kidney-to-body weight ratio was increased approximately two-fold in DN mice, which was compared to control mice. However, there was no change by the extract treatment (Figure A1C). The increased urinary volume in DN mice was also not significantly affected by the extract treatment (Figure A1D). These results indicate that both D and H extracts improve the renal filtration function and protect from DN-induced renal injury. The leaf extracts, similar to flower extracts, exerted a reno-protective effect.

### 3.2. AM Extracts Attenuated Glomerular and Tubular Damage in DN Mice

**Figure 2:**
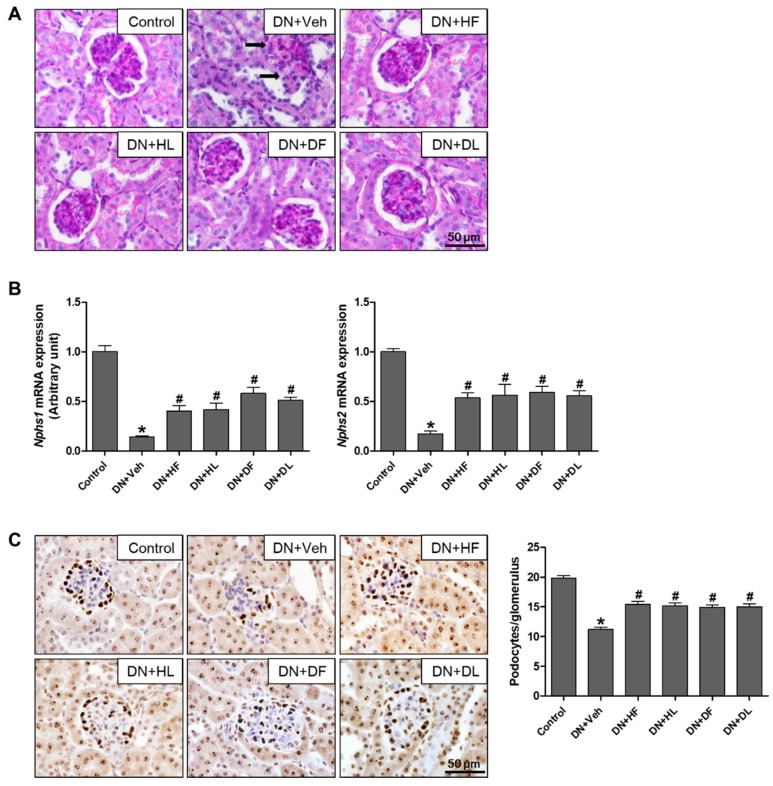
AM extract treatment reduced glomerular lesions in DN mice. Kidney tissues were processed for PAS staining and representative images are shown (**A**). Arrows indicate damaged glomeruli. Relative mRNA expression levels of Nphs1 and Nphs2 were determined by real-time PCR analysis (**B**). Kidney tissues were processed for WT1 immunostaining, representative images were shown, and the number of stained podocyte per glomerulus was counted (**C**). The data are presented as the mean ± SEM. * *p* < 0.05 versus control, # *p* < 0.05 versus DN mice. Scale bar, 50 μm.

**Figure 3:**
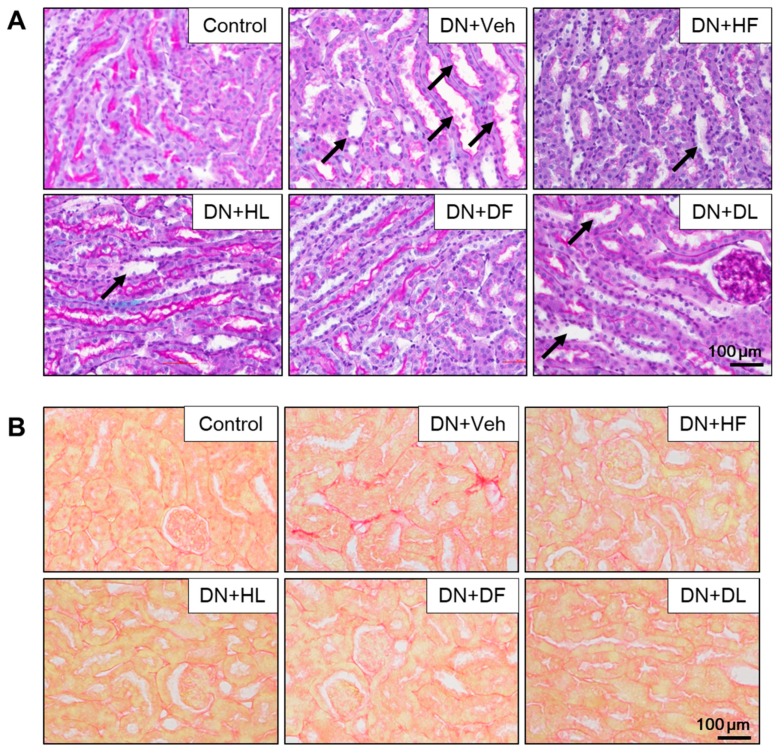
AM extract treatment reduced tubular damage and collagen accumulation in DN mice. Kidney tissues were processed for PAS (**A**) and Sirus red staining (**B**). Representative images are shown. Arrows indicate damaged tubules. Scale bar, 100 μm.

To examine histological changes, renal sections were stained with PAS solution, which is shown in Figure 2A. DN mice showed a severely damaged glomerular structure, which demonstrated diffuse mesangial matrix expansion and thickening of the glomerular basement membrane. The extract treatment decreased the glomerular damage and protected from podocyte loss, which is shown by a significant increase in expressions of *Nphs1* (nephrin) and *Nphs2* (podocin), and the podocyte markers being reduced in DN mice (Figure 2B). Consistently, the extract-treated DN mice showed a significant increase in the number of podocytes/glomerulus as shown by WT1 immunolocalization (Figure 2C). These results indicate that the extract treatment protects DN mice from glomerular lesions and podocyte loss. In addition to glomerular lesions, DN mice showed tubular dilation and degeneration by PAS staining (Figure 3A) and interstitial fibrosis by Sirius red staining (Figure 3B). The extract treatment reduced the tubular damage and collagen accumulation in DN mice, which suggests that both D and H extracts protect DN kidney from structural and functional damage.

### 3.3. AM Extracts Improved Autophagy and Mitochondrial Regulation Altered in the Kidney of DN Mice

**Figure 4:**
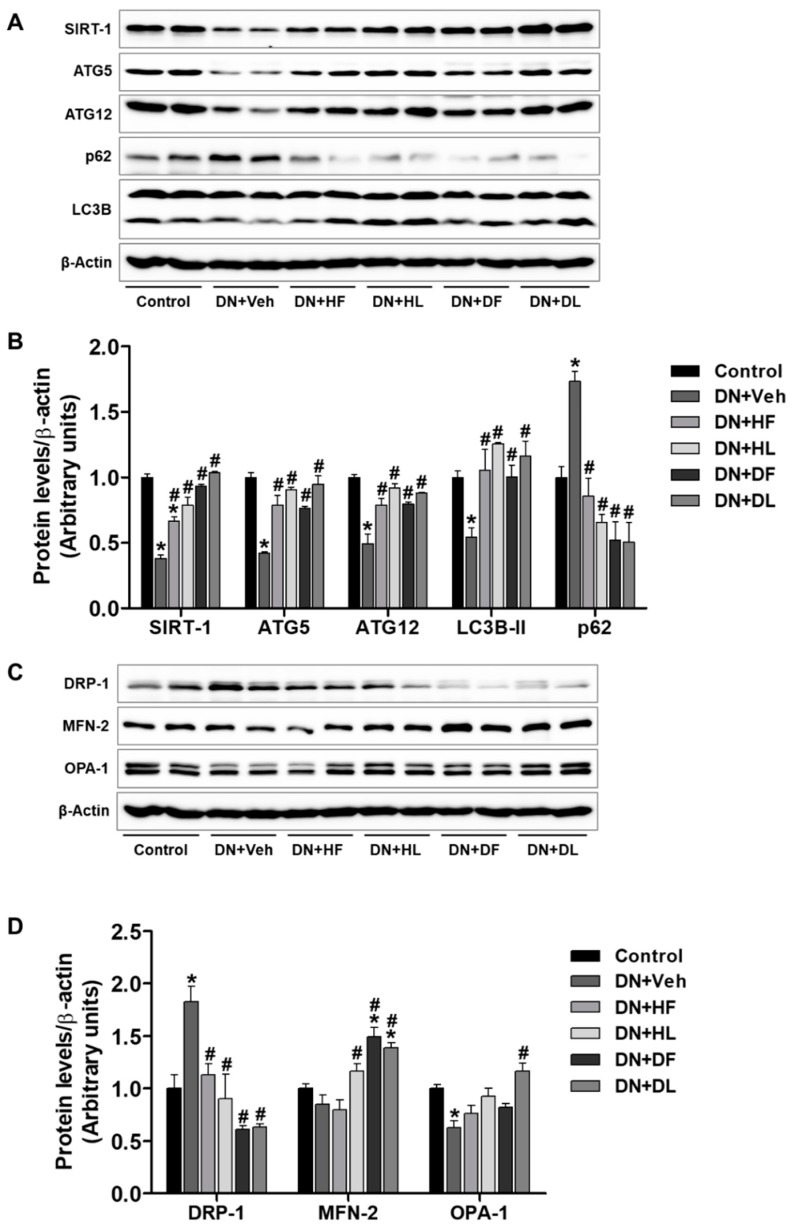
AM extract treatment changed renal expression of autophagy and mitochondrial regulatory proteins in DN mice. Kidney tissues were lysed for Western blot analysis and protein expression levels of autophagy proteins (SIRT-1, ATG5, ATG12, p62, and LC3B). Mitochondrial regulatory proteins (DRP-1, MFN-2, and OPA-1) are shown. The quantitative analysis is included. Western analyses on autophagy (**A**,**B**) and mitochondrial regulatory proteins (**C**,**D**) are shown. The data are presented as the mean ± SEM. * *p* < 0.05 versus the control, # *p* < 0.05 versus DN mice.

Diabetic kidneys were defective in autophagy activity and the podocyte-specific or proximal tubule-specific autophagy-deficient mice were susceptible to acute and chronic kidney injuries [18]. The autophagy activity is regulated by SIRT-1 whose expression is inversely correlated with the pathology and progression of diabetic kidney disease [19]. We, therefore, examined the expression of SIRT-1, ATGs, and autophagy markers, LC3B and p62, in the kidney of control and vehicle-treated or extract-treated DN mice (Figure 4). The extract treatment increased the expression of SIRT-1, ATG5, and ATG12 in DN mice when compared to the vehicle treatment. The extract treatment also improved autophagy dynamics, which is shown by the increased LC3B-II levels and the decreased p62 levels in DN mice (Figure 4A,B). These results suggest that both D and H extracts increase autophagy activity in DN mice.

Mitochondrial dysfunction and oxidative stress are involved in metabolic disorders and mitochondrial dynamics are impaired in Type 2 diabetes [20]. We, therefore, examined the expression of proteins regulating mitochondrial fission (DRP-1) and fusion (MFN-2 and OPA-1). The DRP-1 levels were decreased by all treatments, MFN-2 levels were increased by HL, DF, and DL treatments, and OPA-1 levels were increased by the DL treatment (Figure 4C,D). The results suggest that excessive mitochondrial fragmentation has been attenuated in the kidney of extract-treated DN mice.

### 3.4. AM Extracts Attenuated Hepatic Damage and Lipid Accumulation in DN Mice

**Figure 5:**
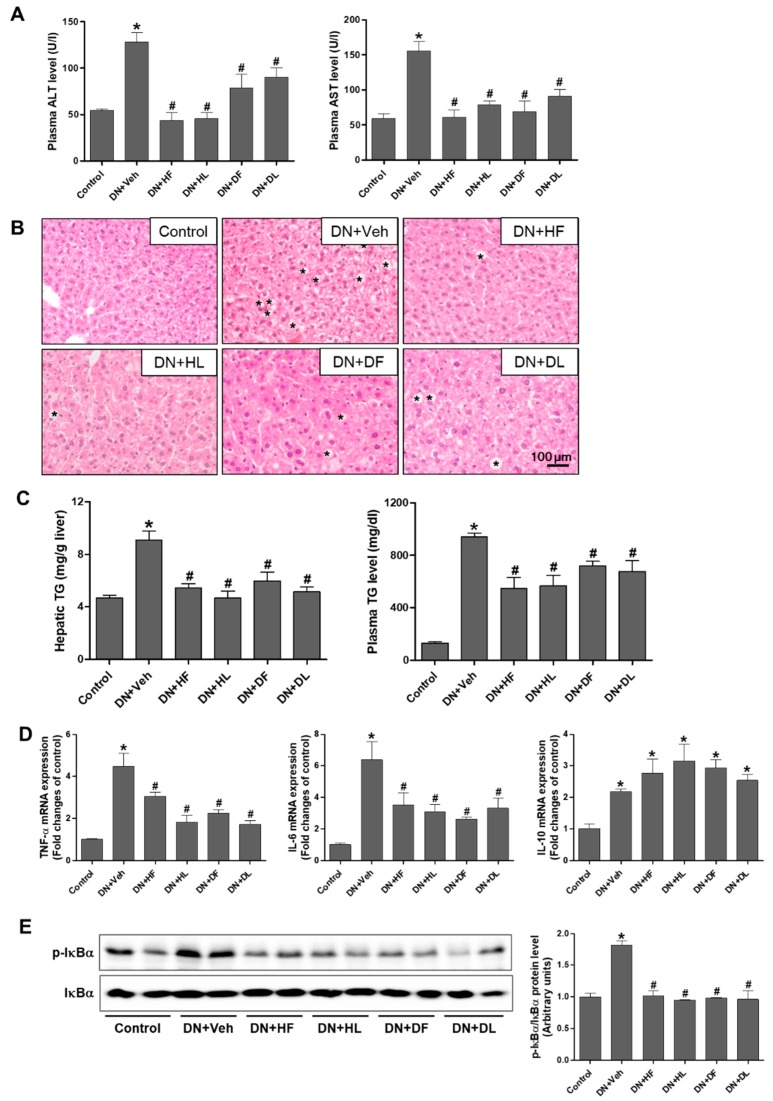
AM extract treatment reduced hepatic injury and lipid accumulation in DN mice. Blood and liver samples were collected from control, vehicle-treated or extract-treated DN mice and plasma ALT and AST (**A**) were measured. Liver tissues were processed for H&E staining and representative images are shown (**B**). Stars indicate lipid droplets. The levels of hepatic and plasma triglycerides were determined in hepatic lysates and plasma samples (**C**). Relative mRNA expression levels of TNFα, IL-6, and IL-10 were determined by real-time PCR analysis (**D**). Liver tissues were lysed for Western blot analysis and protein expression levels of p-IκB(α), IκB(α), and β-actin are shown. The quantitative analysis is included (**E**). The data are presented as the mean ± SEM. * *p* < 0.05 versus control, # *p* < 0.05 versus DN mice. Scale bar, 100 μm.

Diabetic livers are defective in glucose and lipid homeostasis. Therefore, we examined whether the extract treatment attenuates hepatic damage in DN mice. The hepatic injury was assessed by plasma ALT and AST levels (Figure 5A). The increased ALT and AST levels in DN mice were significantly reduced by the extract treatment. H&E staining of liver sections showed a significant increase in hepatic necrosis and lipid accumulation in DN mice, which was reduced by the extract treatment (Figure 5B). Consistently, the levels of hepatic and plasma triglycerides were increased in DN mice and significantly reduced by the extract treatment (Figure 5C).

Because an excessive lipid accumulation exerts inflammatory responses in fatty livers, proinflammatory cytokine levels were measured to assess the changes by the extract treatment in DN mice (Figure 5D). The mRNA expression levels of the tumor necrosis factor alpha (TNF-α) and interleukin-6 (IL-6) were significantly increased in DN mice and the levels were reduced by the extract treatment. The anti-inflammatory IL-10 levels were increased in DN mice but were not changed by the extract treatment. To investigate the effect on NF-κB-mediated inflammatory response by the extracts, we performed Western blotting of phosphorylation of IκBα (Ser32). As shown in Figure 5E, p-IκBα levels were increased in DN mice when compared to the control and significantly reduced by the extract treatment. These results indicate that both D and H extracts have attenuated hepatic lipid accumulation and inflammatory responses in DN mice.

### 3.5. AM Extracts Increased Autophagy Protein Expression in the Liver of DN Mice

**Figure 6:**
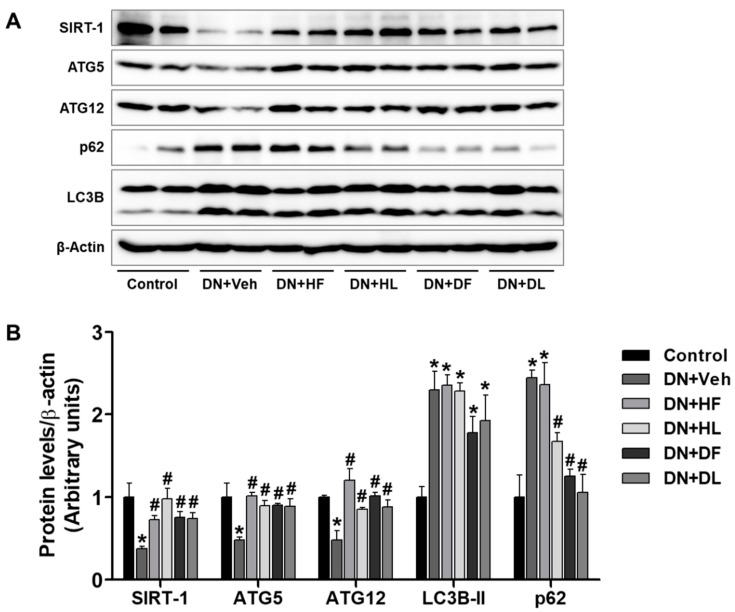
AM extract treatment changed hepatic expression of autophagy proteins in DN mice. Hepatic tissues were lysed for Western blot analysis and protein expression levels of SIRT-1, ATG5, ATG12, p62, and LC3B, which are shown (**A**). The quantitative analysis is included (**B**). The data are presented as the mean ± SEM. * *p* < 0.05 versus control, # *p* < 0.05 versus DN mice.

Excessive lipid accumulation in diabetes is mostly due to an altered lipid metabolism. However, the hepatic expression of proteins regulating lipogenesis and lipolysis were not changed by the extract treatment in this study. We examined the hepatic expressions of SIRT-1, ATG5, ATG12, LC3B, and p62 in control and vehicle-treated or extract-treated DN mice (Figure 6). The extract treatment increased the levels of SIRT-1, ATG5, and ATG12 in DN mice compared to the vehicle treatment. The autophagy dynamics were shown by LC3B-II and p62 levels. LC3B-II levels were significantly increased in DN mice compared to control and similarly increased in the extract-treated DN mice. However, p62 levels were significantly decreased by DL, HF, and HL treatments. These results suggest that the extract treatment increases autophagy activity, which may contribute to the removal of accumulated hepatic lipid droplets.

## 4. Discussion

In this study, we examined the therapeutic effects of AM extracts in an early stage of DN. The flower or leaf extracts from Dakpul and Huangshukuihua (Korean and Chinese subspecies of AM, respectively) were administered orally to DN mice. We found that both D and H extracts increased renal infiltration rates and decreased glomerular and tubular injury and collagen accumulation. The extracts also reduced hepatic injury and lipid accumulation in DN mice. We hypothesize that the improved autophagy activity and mitochondrial function may be an underlying molecular mechanism of the AM effects. The extract treatment increased the expression of autophagy proteins and mitochondrial fusion versus fission and may prevent the accumulation of damaged proteins (e.g., fibrotic or lipid proteins) and mitochondrial dysfunction.

We developed a DN mouse model through a combined treatment of HFD (60% kcal) and streptozotocin (100 mg/kg) injection after unilateral nephrectomy and analyzed the effects of AM extracts after 5 weeks of treatments. C57BL/6 mice are widely used for transgenic mouse models but are relatively resistant to DN when compared to other strains. Genetic modifications (e.g., eNOS-/-, leptin resistant db/db) and STZ treatment accelerate the renal injury by contributing to hyperglycemia and glomerular hyperfiltration [21]. A recent report has shown that combining STZ and unilateral nephrectomy (UN) is an effective method for inducing DN in C57BL/6 mice [22]. HFD has shown to provide a pro-fibrotic effect on UN or STZ treated C57BL/6 mice, which is critical for the progression of chronic kidney diseases (CKD) [23,24]. In this study, we developed a DN model by combining HFD, UN, and STZ to induce DN for a relatively short time (less than 4 weeks). It is an accelerated type 2 diabetic nephropathy model. We treated the extracts at early stages of disease for delaying the progression of DN. Compared to other diabetic kidney injury models, our experimental model is suitable for studying the early pathology of DN [21,25]. Currently, the control of hyperglycemia, dyslipidemia, and blood pressure and use of RAS inhibitors are known to be effective in the early stages of some DN patients. However, the effects are less apparent in patients with progressed DN. Interventions at an early stage are also beneficial for reducing medical expenses and treatment duration [26]. We particularly focused on the autophagy and mitochondrial dysfunction at early stages of DN and investigated the effects of AM extracts.

The AM extracts have previously been shown to decrease urinary albumin excretion by preventing glomerular podocyte apoptosis in streptozotocin-induced DN rats especially through their active component hyperoside [27]. The AM extracts also attenuated oxidative stress and renal fibrosis in streptozotocin-induced DN rats by regulating p38 mitogen-activated protein kinase (p38MAPK)/serine-threonine kinase (Akt) signaling pathways in the kidneys of DN rats [28]. By activating peroxisome proliferator-activated receptor (PPAR), the AM extracts improved lipid metabolic disorders and ameliorated renal inflammation and glomerular injury in DN rats [14]. However, the effects of AM extracts on autophagy and mitochondrial dynamics have not been investigated.

Kidneys are highly metabolic and mitochondria-rich organs and hyperglycemia-induced mitochondrial fragmentation is often observed in the early stages of DN. The mitochondrial structure was severely damaged in HFD-induced diabetic kidneys where proximal tubular mitochondria were small and disorganized often with few cristae structures. A cardiolipin-targeting peptide that protects mitochondrial structure prevented glomerular and proximal tubular injury [29]. The expression of mitochondrial fission-fusion proteins was also altered in DN and the amelioration of early abnormalities in mitochondrial morphology may effectively delay the DN progression [30]. Podocyte-specific *Drp-1* deletion in diabetic mice decreased DN pathology through enhanced mitochondrial function [31]. MFN-2 overexpression improved kidney function in streptozotocin-induced diabetic rats [32]. In our study, extract-treated DN mice showed a decrease of DRP-1. However, an increase of MFN-2 and OPA-1 suggests that an inhibition of mitochondrial fragmentation by AM extracts may improve mitochondrial function in DN mice and attenuate the progression of the disease.

Autophagy is an initial adaptive response to metabolic stress and insufficient autophagy, which may lead to DN pathology [33]. Recent studies reported that podocyte-specific *Atg5*-deficient mice developed massive proteinuria under HFD conditions where excessive accumulation of p62 and damaged autophagy-lysosome structures were found [6]. The proteinuria-induced tubulointerstitial lesions were also exacerbated in proximal tubule-specific *Atg5*-deficient mice [34]. In our study, extract-treated DN mice showed a significant increase of autophagy proteins (ATG5, ATG12, and LC3B-II) and a dramatic decrease of p62, which suggests that enhanced autophagy activity contributes to clearing toxic aggregates and delaying the progression of the disease.

P62 is an autophagy receptor that links ubiquitinated proteins to LC3B-bound autophagosomes that are degraded after lysosomal fusion. Thus, it serves as an index of autophagic degradation. A reporter cell system monitored an autophagic flux in vitro by using the inducible expression of GFP-LC3B or GFP-fused autophagic receptor p62 or NBR1. The GFP-p62 was most efficiently degraded on starvation and performed the best [35]. The autophagic flux has also been monitored by using LC3 and p62 tagged to a tandem fusion of acid-insensitive mCherry and acid-sensitive GFP to distinguish easily autophagosomes from autolysosomes [36]. The live imaging of the double tag is extremely informative about the autophagic process. In our study, extract-treated DN mice showed a dramatic decrease of p62 reflecting upregulated autophagic clearance. Furthermore, p62 is a multifaceted receptor that performs diverse functions such as amino acid sensing and the oxidative stress response [37]. The loss of *p62* decreased ubiquitin-positive aggregates and markedly attenuated liver injury in *Atg7*-deficient mice [38]. P62 accumulation has shown to activate the transcription factor and the nuclear factor erythroid 2-related factor 2 (Nrf2) through the direct interaction with Kelch-like ECH-associated protein 1 (Keap1) [39,40]. Therefore, p62 functions as an autophagy receptor as well as a signaling hub. It is critical to maintain homeostatic levels of p62.

Recently, autophagy has shown to regulate the accumulation of fibrogenic proteins. Activation of autophagy promotes the degradation of transforming growth factor (TGF)-β and its secretion into interstitial cells and suppresses renal fibrosis in an in vivo model of chronic kidney disease [7]. Autophagy-deficient fibroblast cells were impaired in the degradation of internalized collagen and distribution of focal adhesions that may increase the risk of developing fibrosis [41]. In this case, we suggest that AM extracts may promote the degradation of fibrogenic proteins by enhancing autophagy activity. However, the exact molecular mechanisms remain to be clarified.

A turnover of lipid droplets (LDs) through autophagy, called lipophagy, is important for supplying free fatty acids hydrolyzed from TGs to produce energy during nutrient deprivation. LDs have shown to be associated with autophagosomes in fasted mice and inhibition of autophagy resulted in increased TG and LDs [42]. The molecular mechanisms of selective autophagy or adaptors for LDs have been proposed [43,44], which suggests a cooperation between lipophagy and lipolysis through adipose triglyceride lipase (ATGL) and SIRT1 as a key mediator. Autophagic dysfunction is associated with steatosis, insulin resistance, and hepatic inflammation in obese mice and patients with non-alcoholic fatty liver disease [45,46,47]. The pharmacological activation of autophagy may alleviate lipotoxic effects by preventing lipid accumulation and facilitate metabolic adaptation of the liver to metabolic stresses [47]. In this study, we suggest that the increased autophagy activity by AM extracts contributes to the removal of LDs and improve the associated pathology.

Our results strongly support that developing drugs to improve autophagy activity is a promising therapeutic strategy for DN [20,48]. Recently, several drugs were reported to attenuate podocyte dysfunction and DN progression by enhancing upstream regulators of autophagy in mouse and rat DN models [49,50]. In addition, an autophagy regulatory network by using a systems-level bioinformatics study is available to search novel transcription factors or microRNAs key to the control of autophagy [51]. The AM extracts used in this study are a mixture of bioactive components obtained from D or H. Therefore, further study is necessary to find effective components of each extract on autophagy processes to develop new drugs for clinical trials.

In summary, we demonstrated that AM extracts from both D and H attenuated renal injury, glomerular and tubular damage, and collagen accumulation of DN mice. We also found that the AM extracts reduced hepatic injury and lipid accumulation of DN mice. In this study, we provided a new molecular mechanistic insight that AM extracts improve autophagy activity and mitochondrial function to attenuate the DN progression.

## References

[1] Jefferson J.A., Shankland S.J., Pichler R.H. (2008). Proteinuria in diabetic kidney disease: A mechanistic viewpoint. Kidney Int..

[2] Kim Y., Park C.W. (2017). New therapeutic agents in diabetic nephropathy. Korean J. Intern. Med..

[3] Rabinowitz J.D., White E. (2010). Autophagy and metabolism. Science.

[4] Kume S., Koya D. (2015). Autophagy: A novel therapeutic target for diabetic nephropathy. Diabetes Metab. J..

[5] Kume S., Koya D., Uzu T., Maegawa H. (2014). Role of nutrient-sensing signals in the pathogenesis of diabetic nephropathy. Biomed. Res. Int..

[6] Tagawa A., Yasuda M., Kume S., Yamahara K., Nakazawa J., Chin-Kanasaki M., Araki H., Araki S., Koya D., Asanuma K. (2016). Impaired podocyte autophagy exacerbates proteinuria in diabetic nephropathy. Diabetes.

[7] Ding Y., Kim S., Lee S.Y., Koo J.K., Wang Z., Choi M.E. (2014). Autophagy regulates TGF-beta expression and suppresses kidney fibrosis induced by unilateral ureteral obstruction. J. Am. Soc. Nephrol..

[8] Kume S., Yamahara K., Yasuda M., Maegawa H., Koya D. (2014). Autophagy: Emerging therapeutic target for diabetic nephropathy. Semin. Nephrol..

[9] Youle R.J., van der Bliek A.M. (2012). Mitochondrial fission, fusion, and stress. Science.

[10] Ni H.M., Williams J.A., Ding W.X. (2015). Mitochondrial dynamics and mitochondrial quality control. Redox. Biol..

[11] Yu T., Jhun B.S., Yoon Y. (2011). High-glucose stimulation increases reactive oxygen species production through the calcium and mitogen-activated protein kinase-mediated activation of mitochondrial fission. Antioxid. Redox. Signal..

[12] Useful Tropical Plants Database. http://tropical.theferns.info/.

[13] Yang G., Zhang M., Zhang M., Chen S., Chen P. (2015). Effect of huangshukuihua (flos abelmoschi manihot) on diabetic nephropathy: A meta-analysis. J. Tradit. Chin. Med..

[14] Ge J., Miao J.J., Sun X.Y., Yu J.Y. (2016). Huangkui capsule, an extract from *Abelmoschus manihot* (L.) medic, improves diabetic nephropathy via activating peroxisome proliferator-activated receptor (PPAR)-alpha/gamma and attenuating endoplasmic reticulum stress in rats. J. Ethnopharmacol..

[15] Xue C., Guo J., Qian D., Duan J.A., Shang E., Shu Y., Lu Y. (2011). Identification of the potential active components of *Abelmoschus manihot* in rat blood and kidney tissue by microdialysis combined with ultra-performance liquid chromatography/quadrupole time-of-flight mass spectrometry. J. Chromatogr. B Anal. Technol. Biomed. Life Sci..

[16] Ahn K. (2017). The worldwide trend of using botanical drugs and strategies for developing global drugs. BMB Rep..

[17] Jung S., Son H., Hwang C.E., Cho K.M., Park S.W., Kim H.J. (2018). Ganoderma lucidum ameliorates non-alcoholic steatosis by upregulating energy metabolizing enzymes in the liver. J. Clin. Med..

[18] Ding Y., Choi M.E. (2015). Autophagy in diabetic nephropathy. J. Endocrinol..

[19] Yacoub R., Lee K., He J.C. (2014). The role of sirt1 in diabetic kidney disease. Front Endocrinol..

[20] Rovira-Llopis S., Banuls C., Diaz-Morales N., Hernandez-Mijares A., Rocha M., Victor V.M. (2017). Mitochondrial dynamics in type 2 diabetes: Pathophysiological implications. Redox. Biol..

[21] Betz B., Conway B.R. (2014). Recent advances in animal models of diabetic nephropathy. Nephron. Exp. Nephrol..

[22] Uil M., Scantlebery A.M.L., Butter L.M., Larsen P.W.B., de Boer O.J., Leemans J.C., Florquin S., Roelofs J. (2018). Combining streptozotocin and unilateral nephrectomy is an effective method for inducing experimental diabetic nephropathy in the ‘resistant’ C57Bl/6J mouse strain. Sci. Rep..

[23] Gai Z., Hiller C., Chin S.H., Hofstetter L., Stieger B., Konrad D., Kullak-Ublick G.A. (2014). Uninephrectomy augments the effects of high fat diet induced obesity on gene expression in mouse kidney. Biochim. Biophys. Acta.

[24] Kim D.H., Choi B.H., Ku S.K., Park J.H., Oh E., Kwak M.K. (2016). Beneficial effects of sarpogrelate and rosuvastatin in high fat diet/streptozotocin-induced nephropathy in mice. PLoS ONE.

[25] Alpers C.E., Hudkins K.L. (2011). Mouse models of diabetic nephropathy. Curr. Opin. Nephrol. Hypertens..

[26] Kim S.S., Kim J.H., Kim I.J. (2016). Current challenges in diabetic nephropathy: Early diagnosis and ways to improve outcomes. Endocrinol. Metab..

[27] Zhou L., An X.F., Teng S.C., Liu J.S., Shang W.B., Zhang A.H., Yuan Y.G., Yu J.Y. (2012). Pretreatment with the total flavone glycosides of flos *Abelmoschus manihot* and hyperoside prevents glomerular podocyte apoptosis in streptozotocin-induced diabetic nephropathy. J. Med. Food.

[28] Mao Z.M., Shen S.M., Wan Y.G., Sun W., Chen H.L., Huang M.M., Yang J.J., Wu W., Tang H.T., Tang R.M. (2015). Huangkui capsule attenuates renal fibrosis in diabetic nephropathy rats through regulating oxidative stress and p38mapk/akt pathways, compared to alpha-lipoic acid. J. Ethnopharmacol..

[29] Szeto H.H., Liu S., Soong Y., Alam N., Prusky G.T., Seshan S.V. (2016). Protection of mitochondria prevents high-fat diet-induced glomerulopathy and proximal tubular injury. Kidney Int..

[30] Forbes J.M., Thorburn D.R. (2018). Mitochondrial dysfunction in diabetic kidney disease. Nat. Rev. Nephrol..

[31] Ayanga B.A., Badal S.S., Wang Y., Galvan D.L., Chang B.H., Schumacker P.T., Danesh F.R. (2016). Dynamin-related protein 1 deficiency improves mitochondrial fitness and protects against progression of diabetic nephropathy. J. Am. Soc. Nephrol..

[32] Tang W.X., Wu W.H., Zeng X.X., Bo H., Huang S.M. (2012). Early protective effect of mitofusion 2 overexpression in STZ-induced diabetic rat kidney. Endocrine.

[33] Gonzalez C.D., Lee M.S., Marchetti P., Pietropaolo M., Towns R., Vaccaro M.I., Watada H., Wiley J.W. (2011). The emerging role of autophagy in the pathophysiology of diabetes mellitus. Autophagy.

[34] Yamahara K., Kume S., Koya D., Tanaka Y., Morita Y., Chin-Kanasaki M., Araki H., Isshiki K., Araki S., Haneda M. (2013). Obesity-mediated autophagy insufficiency exacerbates proteinuria-induced tubulointerstitial lesions. J. Am. Soc. Nephrol..

[35] Larsen K.B., Lamark T., Overvatn A., Harneshaug I., Johansen T., Bjorkoy G. (2010). A reporter cell system to monitor autophagy based on p62/SQSTM1. Autophagy.

[36] Pankiv S., Clausen T.H., Lamark T., Brech A., Bruun J.A., Outzen H., Overvatn A., Bjorkoy G., Johansen T. (2007). P62/SQSTM1 binds directly to Atg8/LC3 to facilitate degradation of ubiquitinated protein aggregates by autophagy. J. Biol. Chem..

[37] Katsuragi Y., Ichimura Y., Komatsu M. (2015). P62/SQSTM1 functions as a signaling hub and an autophagy adaptor. FEBS J..

[38] Komatsu M., Waguri S., Koike M., Sou Y.S., Ueno T., Hara T., Mizushima N., Iwata J., Ezaki J., Murata S. (2007). Homeostatic levels of p62 control cytoplasmic inclusion body formation in autophagy-deficient mice. Cell.

[39] Jain A., Lamark T., Sjottem E., Larsen K.B., Awuh J.A., Overvatn A., McMahon M., Hayes J.D., Johansen T. (2010). P62/SQSTM1 is a target gene for transcription factor NRF2 and creates a positive feedback loop by inducing antioxidant response element-driven gene transcription. J. Biol. Chem..

[40] Komatsu M., Kurokawa H., Waguri S., Taguchi K., Kobayashi A., Ichimura Y., Sou Y.S., Ueno I., Sakamoto A., Tong K.I. (2010). The selective autophagy substrate p62 activates the stress responsive transcription factor Nrf2 through inactivation of Keap1. Nat. Cell Biol..

[41] Kawano S., Torisu T., Esaki M., Torisu K., Matsuno Y., Kitazono T. (2017). Autophagy promotes degradation of internalized collagen and regulates distribution of focal adhesions to suppress cell adhesion. Biol. Open.

[42] Singh R., Kaushik S., Wang Y., Xiang Y., Novak I., Komatsu M., Tanaka K., Cuervo A.M., Czaja M.J. (2009). Autophagy regulates lipid metabolism. Nature.

[43] Kaushik S., Cuervo A.M. (2015). Degradation of lipid droplet-associated proteins by chaperone-mediated autophagy facilitates lipolysis. Nat. Cell Biol..

[44] Sathyanarayan A., Mashek M.T., Mashek D.G. (2017). ATGL promotes autophagy/lipophagy via SIRT1 to control hepatic lipid droplet catabolism. Cell Rep..

[45] Fukuo Y., Yamashina S., Sonoue H., Arakawa A., Nakadera E., Aoyama T., Uchiyama A., Kon K., Ikejima K., Watanabe S. (2014). Abnormality of autophagic function and cathepsin expression in the liver from patients with non-alcoholic fatty liver disease. Hepatol. Res..

[46] Yang L., Li P., Fu S., Calay E.S., Hotamisligil G.S. (2010). Defective hepatic autophagy in obesity promotes ER stress and causes insulin resistance. Cell Metab..

[47] Ueno T., Komatsu M. (2017). Autophagy in the liver: Functions in health and disease. Nat. Rev. Gastroenterol. Hepatol..

[48] Rubinsztein D.C., Codogno P., Levine B. (2012). Autophagy modulation as a potential therapeutic target for diverse diseases. Nat. Rev. Drug Discov..

[49] Jin Y., Liu S., Ma Q., Xiao D., Chen L. (2017). Berberine enhances the AMPK activation and autophagy and mitigates high glucose-induced apoptosis of mouse podocytes. Eur. J. Pharmacol..

[50] Wang X., Gao L., Lin H., Song J., Wang J., Yin Y., Zhao J., Xu X., Li Z., Li L. (2018). Mangiferin prevents diabetic nephropathy progression and protects podocyte function via autophagy in diabetic rat glomeruli. Eur. J. Pharmacol..

[51] Turei D., Foldvari-Nagy L., Fazekas D., Modos D., Kubisch J., Kadlecsik T., Demeter A., Lenti K., Csermely P., Vellai T. (2015). Autophagy regulatory network—A systems-level bioinformatics resource for studying the mechanism and regulation of autophagy. Autophagy.

